# Mitochondria at the extremes: pioneers, protectorates, protagonists

**DOI:** 10.1186/2046-7648-3-10

**Published:** 2014-05-12

**Authors:** Andrew J Murray

**Affiliations:** 1 Department of Physiology, Development and Neuroscience, University of Cambridge, Downing Street, Cambridge CB2 3EG, UK

## Abstract

The engulfment of a proto-mitochondrion by a primitive unicellular organism gave rise to the first eukaryotic cell, and ever since, mitochondrial function has been a vital aspect of eukaryotic life. Under conditions of physiological stress, the mitochondrion is far from a passive bystander, instead playing a key role in signalling pathways and the cellular responses they elicit. In this thematic series of *Extreme Physiology & Medicine*, the role of the mitochondrion in the response to physiological stress will be considered anew, through research articles, reviews, viewpoints and methodology papers that aim to reposition the organelle as a key player in the human response to a wide range of extreme conditions.

## Pioneers

The endosymbiotic theory holds that a pioneering proto-mitochondrion entered a primitive host cell, taking up residence in the intracellular environment and thereby laying the foundations of eukaryotic life. It remains a matter of debate as to whether the act of engulfment that followed this initial coming together constituted a conquest on the part of the larger organism or an invasion by its parasitic intruder [1], but a realignment of responsibilities followed which ensured that this became a relationship of mutual benefit. The mitochondrion surrendered much of its genetic material, and thus executive control to the host cell, whilst the host delegated a number of key functions to the nascent organelle, chiefly energy production *via* oxidative phosphorylation, but also catabolic and anabolic processes, apoptotic cell death and, to a degree, sex determination [2]. Retaining a limited autonomy, represented by a single circular DNA plasmid and distinct ribosomes, the mitochondria had become protectorate states of the imperial host, with intracellular specialisation becoming feature of the new regime.

To the host cell, the benefits of this union were clear. The mitochondrion, with its highly invaginated inner membrane replete with electron carriers and proton pumps, ramped up the capacity of the newly chimaeric organism for ATP synthesis, earning the mitochondria their often-used sobriquet: *the powerhouses of the cell*. The chemiosmotic coupling of fuel oxidation to ADP phosphorylation far exceeded the extent to which the host could previously extract free energy from substrates via fermentation processes, and thereby fuelled the generation of complex life, that eventually led to the development of true multicellularity: tissues, organs and systems.

## Protectorates

Cushioned within the homeostatically controlled confines of the eukaryotic cell, the modern mitochondrion normally enjoys a somewhat comfortable existence. In exchange for meeting the host organism’s ATP requirements, highly adapted systems of gas exchange, nutrient acquisition and transport ensure that optimal conditions, including constant oxygen and substrate provision, are as far as possible maintained in the face of fluctuating environmental factors, pathology and physiological stress. Indeed, the extent to which an organism can acclimatise to such extreme challenges that might otherwise threaten homeostasis defines the limits of its tolerance. Eukaryotes typically lack the biochemical richness and diverse metabolic pathways that have allowed some prokaryotes, the so-called *extremophiles*, to specialise to true extremes of temperature, pH or osmotic pressure and hence thrive under conditions that would be detrimental to most life on Earth. Nevertheless, a great challenge faces eukaryotes under non-optimal conditions, in the need to maintain energy metabolism, and this is particularly acute in those animals that practise endothermy, the birds and mammals, which thus have a greatly elevated metabolic rate in comparison to other organisms.

In man, a classic view of acclimatisation to the extremes might be limited to gross physiological responses that attempt to maintain a constant intracellular environment. In the hypobaric hypoxia of high altitude, for instance, an erythropoietic response elevates haematocrit such that arterial oxygen content is maintained in the face of decreased haemoglobin-oxygen saturation [3], whilst coordinated ventilatory and cardiovascular responses act to maintain oxygen delivery [4]. So is the protectorate mitochondrion, encased within its guardian cell, thereby immune to such a fall barometric pressure, with the limitations in supply of a vital commodity perfectly compensated by adaptations in oxygen delivery alone? To a certain extent, this might be the case, but a growing awareness of the intricate cellular response to hypoxia suggests that modifications to oxygen utilisation, including coordinated inhibition of protein synthesis (as a means to decrease demand), down-regulation of mitochondrial oxidative phosphorylation, substrate switching and, in some tissues, loss of mitochondrial density, accompany attempts to maintain supply [5,6]. Lowering oxygen consumption can thus elevate cellular oxygen tension (pO_2_) such that the remaining mitochondria might function more effectively. Central to the cellular response to hypoxia are the hypoxia-inducible factor (HIF) transcription factors, which are stabilised under conditions of low pO_2_ and modify the expression of genes that underlie all aspects of the human hypoxic response [7]. The mitochondria may also act as the nexus of a further feedback loop, generating increased reactive oxygen species (ROS) under hypoxic conditions, which themselves stabilise HIF, leading to a rebalancing of oxygen supply and demand and thus preventing further excess ROS generation thereby mitigating oxidative damage [6]. In the case of hypoxia then, the mitochondria are more than simple bystanders and might be considered protagonists, acting not as mere end consumers of a constantly maintained oxygen supply but playing key roles in both the cellular sensing of fluctuating oxygen levels and the consequent response to limited supply.

## Protagonists

In this thematic series of *Extreme Physiology* & *Medicine*, the role of the mitochondrion in the response to physiological stress will be considered anew, through research articles, reviews, viewpoints and methodology papers that might aim to reposition the organelle as a key player in the human response to a wide range of extreme conditions. Indeed, whilst the mitochondrion emerges as a central component of the cell’s hypoxic response, reprogramming of metabolic pathways also underlies the response to limitations in substrate supply during short-term fasting and longer-term starvation. In times of plenty, excess reduced carbon in the form of glucose is converted to a longer-term storage form of triglycerides and sequestered in adipose tissue in order to ensure that substrate supply can continue through leaner times, and this relies heavily on mitochondrial pathways of *de novo* lipogenesis in the liver and/or adipose tissue itself [8]. Under fasting conditions, metabolically omnivorous tissues, such as cardiac muscle, switch mitochondrial substrate preference from limited pyruvate towards plentiful fatty acid reserves, via enzyme phosphorylation/inhibition and modification of metabolic gene expression [8]. Meanwhile, during longer-term starvation, the liver meets the brain’s demand for non-fatty acid substrates via mitochondrial-led ketogenesis [9].

During endurance events, skeletal muscle mitochondrial density is a determinant of exercise capacity, with training stimulating mitochondrial biogenesis, and conversely, periods of inactivity, during injury lay-off, space flight or prolonged bed rest for instance, driving a coordinated detraining response and consequent loss of fitness that is characterised by a fall in muscle mitochondrial content [10]. Finally, during cold acclimation, mitochondrial uncoupling offers a route towards non-shivering thermogenesis, albeit at the cost of inefficient ATP production. Most effective, in this regard, is brown adipose tissue—once thought to be the preserve of hibernating species and human infants, but now known to exist in an active form in many adult humans too [11].

From the initial incorporation of the proto-mitochondrion into the first host cell—the event that gave rise to complex eukaryotic life—it follows that mitochondrial function is central to all aspects of human physiology. Indeed, preservation of mitochondrial function is quite literally vital to our survival, having been shown to correlate positively with outcome in the critically ill [12]. Moreover, even mitochondrial apoptotic pathways, whilst instigating the controlled death of an individual cell, act to preserve function in the tissue or organism as a whole. It is perhaps, therefore, paradoxical to consider that this organelle, deemed to be so intricately linked with our own survival, also manages our eventual demise. As a significant possible source of ROS within our cells and their own proteins, lipids and unprotected DNA, such proximate targets of oxidative stress, the mitochondria may play a central role in the process of human ageing and ultimate energetic and functional decline [2]. Mitochondria *in extremis*.

## Abbreviations

HIF: hypoxia-inducible factor; ROS: reactive oxygen species.

## Competing interests

The author declares that he has no competing interests.
